# Characteristics of pain and their relationship to disease activity in UK patients with Behçet’s syndrome: a prospective cohort study

**DOI:** 10.1177/20494637231198200

**Published:** 2023-08-29

**Authors:** K Plant, A Goebel, J Nair, R Moots, L Chadwick, N Goodson

**Affiliations:** 1 Department of Rheumatology, 4595 Liverpool University Hospital NHS Foundation Trust, Liverpool, UK; 2 Faculty of Health and Life Sciences, Liverpool University, Liverpool, UK; 3 Pain Research Institute, 4591 University of Liverpool, Liverpool, UK; 4 Department of Pain Medicine, 195157 The Walton Centre NHS Foundation Trust, Liverpool, UK; 5 Department of Rheumatology, National Behcet’s Centre of Excellence, Liverpool, UK; 6 Research Institute for Sport and Exercise Sciences, Liverpool John Moores University, Liverpool, UK

**Keywords:** chronic pain, fibromyalgia, musculoskeletal pain, pain measurement, pain perception

## Abstract

**Background:**

Behçet’s syndrome (BS) is a rare multi-systemic vasculitis of unknown aetiology. Fibromyalgia syndrome (FMS) is more prevalent in rheumatological conditions such-as BS, than the general population. However, there is limited research into the aetiology and characteristics of pain in BS.

**Objectives:**

To describe the pain characteristics and incidence of FMS in people with BS and investigate their relationship with BS disease activity.

**Methods:**

A cohort study of BS patients attending the Liverpool Behçet's Centre between February 2017 and March 2019. BS was defined using the International Study Group Criteria. BS severity was assessed using the Behçet's Disease Current Activity Form. FMS was determined from consultant diagnosis. Assessments of pain included: Pain Visual Analogue Scale (PVAS), Pain Mannequin, Brief Pain Inventory, EQ-5D-3L and Short Form McGill. Pain and FMS prevalence were compared between high and low disease activity.

**Results:**

90% reported moderate-severe pain with a median PVAS score of 68/100 [38, 81]. 35.6% of participants had FMS and 46.5% experienced generalized pain. 76% of participants with high disease activity reported severe pain, compared to 39.1% with low disease activity (*p* = .003). Pain was more generalised in high disease activity (72%) compared to low disease activity (37.7%) (*p* = .003). FMS was more prevalent in the high disease activity group (52%) than the low disease activity group (29%) (*p* = .04).

**Conclusions:**

This is the first study to explore pain in participants with BS in the United Kingdom. The majority of BS patients experience moderate-severe widespread pain. Severe widespread pain is more prevalent in those with high disease activity. We have demonstrated a relationship between high disease activity, worse pain intensity, and FMS. This paper contributes to the understanding of two conditions which remain to be fully understood, FMS and BS, and generates new hypotheses to describe the interplay between.

## Introduction

Behçet's syndrome (BS) is a rare multi-systemic relapsing and remitting vasculitis of unknown aetiology with hallmark manifestations of mucocutaneous ulcers and skin lesions. Diagnosis is challenging given the broad spectrum of disease features and their relapsing and remitting nature. However, the International Study Group (ISG) criteria^1^ and International Criteria for Behçet’s disease (ICBD)^2^ can be used to support clinical diagnosis.

The highest prevalence of BS is across the ‘Silk road’: Middle East and Far East Asia; The UK prevalence of BS is estimated at 0.64 patients per 100,000 population.^3^ Disease prevalence and severity varies geographically.^4,5^ However, there is a paucity of epidemiological data describing BS patients in the UK.^6–8^

Morbidity is high in BS; patients report poor quality of life, fatigue, and pain.^9,10^ However, there is limited research into the aetiology and characteristics of pain in BS.^11,12^ To our knowledge, there are no studies exploring pain in a UK BS population. Fibromyalgia Syndrome (FMS) is a common cause of widespread musculoskeletal pain and is more prevalent in rheumatological conditions such as BS, than in the general population. In an Iraqi study, 58.9% of BS patients experienced widespread pain, however, only 8.9% met criteria for FMS.^13^ Studies evaluating the relationship between FMS and BS severity are conflicting.^14–16^

There are three National Centres of Excellence for BS in the UK: London, Birmingham, and Liverpool. This prospective cohort study was undertaken to describe the pain experience of patients with BS attending the Liverpool Behçet’s Centre (LBC). We aimed to describe the pain characteristics and incidence of comorbid FMS in this cohort and explore their relationship with BS disease activity.

## Methods

BS patients attending LBC were invited to participate in this study between February 2017 and March 2019. This study was granted ethical approval by the Northwest – Liverpool Central Committee (16/NW/0854). All participants consented for inclusion and for their medical records on the LBC database to be used. The cohort was defined as having ‘Complete BS’ if they satisfied all the ISG Criteria and ‘Incomplete BS’ if they met one criterion plus oral ulcers.^17^ Disease duration was defined as the time from diagnosis to the time of consent. Participants completed questionnaires (Table S1) at an initial clinic appointment (baseline), then at a 6-months appointment and 12-months appointment.

Cohort demographics were collected via a basic demographic questionnaire, a self-reported comorbidities questionnaire, the 0–100 Fatigue Visual Analogue Scale (VAS),^18^ EQ VAS^19^ and the Behçet’s disease quality of life (BDQOL) questionnaire.^20^ FMS prevalence was determined from consultant clinical diagnosis. Alongside the self-reported comorbidities, mental health was assessed using the GAD-7^21^ and PHQ-9.^22^

BS severity was assessed using the Behçet’s disease current activity form (BDCAF),^23^ a self-reported severity score. BDCAF scores were used to place participants into high or low disease activity groups. High and low disease activity were defined as a BDCAF score ≥4 or <4, respectively.^24^ To minimise inflation of BS disease severity in those with FMS, a modified BDCAF (with arthralgia and arthritis components removed) was calculated (mBDCAF).

To describe the pain experience, the 0–100 Pain VAS, Pain Mannequin and the Brief Pain Inventory short form (BPI), and the pain domains of the EQ-5D-3L^19^ were used. Using the American College of Rheumatology (ACR) 2016 definition, widespread pain was assigned if the pain mannequin was shaded in all four quadrants (upper left, upper right, lower left, and lower right) plus axial distribution.^25^ The pain mannequin data were also used to assign a widespread pain index score (WPI).^26^ Descriptive pain terms were assessed using the Short Form McGill Pain Questionnaire 2 (SFMPQ2).^27^

Pain and fatigue were compared between high and low disease activity and between those with and without a concomitant FMS diagnosis using Chi-Square Test of Independence. The mean mBDCAFs were compared between those with and without FMS using a student’s T-Test. Stata Version 14 was used to analyse all data. Missing data were not imputed. *p* values <.05 were considered to indicate statistical significance.

## Results

One hundred one participants with BS were evaluated, 75 women and 26 men with an age range of 19–74 years. The baseline questionnaire results are presented below. The cohort demographics are summarised in Table 1.

**Table 1.** Cohort demographics.

	*n	(mean ± SD)
Age	101	42.9 ± 12.64
BS disease status	101	(n, %) (median, IQR)
Complete BS (ISG criteria)		82, 81.2%
Incomplete BS		19, 18.8%
Duration of BS		3.32 years [0.99,11.5]
High disease activity (BDCAF ≥ 4)	94	25, 26.6%
Low disease activity (BDCAF < 4)	94	69, 73.4%
Co-morbidities	101	(n, %)
Heart disease		11, 10.9%
Angina/Myocardial infarction		6, 5.9%
Stroke		5, 5%
Lung disease		15, 14.9%
Diabetes		7, 6.9%
Ulcer/Stomach disease		16, 15.8%
Hypertension		22, 21.8
Kidney disease		3, 3%
Liver disease		4, 4%
Anaemia/Blood disorder		2, 20%
Osteoarthritis		26, 25.7%
Malignancy		6, 5.9%
Depression		39, 38.6%
Quality of life measures		(mean ± SD)
Fatigue VAS	101	66.1 ± 23.1
EQ VAS	100	48 ± 25.4
BD-QOL	100	15.4 ± 9.4

The table describes the age, disease status, disease activity, self-reported comorbidities, fatigue visual analogue scale scores, and quality of life measures (EQ VAS and BDQOL scores) of the cohort. High and low disease activity were defined as a Behçet’s disease current activity form (BDCAF) score ≥4 or <4, respectively.

*n: number of participants with complete data; BS: Behçet’s Syndrome; BDCAF : Behçet’s disease current activity form; VAS: Visual analogue scale; BD-QOL: Behçet’s disease quality of life.

81% of the cohort met the ISG criteria and were termed ‘Complete BS’. Of those not meeting the ISG criteria all met ‘Incomplete BS’ status. 73% had low disease activity.

The most prevalent self-reported comorbidity was depression (38.6%). However, the prevalence of depression on the PHQ-9 was higher; 86.4% had a PHQ-9 score of 5 or above which corresponds with clinically diagnosable depression. Also 76.9% of the cohort had a GAD-7 score of 5 or above which corresponds with clinically diagnosable anxiety (Table S2). A prominent level of fatigue was observed with a mean Fatigue VAS of 66.1 ± 23.08. The cohort reported impaired quality of life; On average participants selected 15.4 ± 9.4 negative effecting life statements out of 30 on the BD-QOL questionnaire and rated their general state of health (EQ VAS) at 48 ± 25.4 out of 100 (best health).

Pain characteristics baseline data are outlined in Table 2. 90% of the cohort reported moderate to severe pain with a median pain VAS score of 68 [38, 81]. Widespread pain was described by 46.5% of the cohort of which 91.3% had a WPI Score ≥7. Focal pain was also described, with 69.3% reporting back pain. A concomitant diagnosis of FMS was recorded for 35.6% of the cohort (40% of females and 23.1% of males). There were no statistical differences in pain characteristics between genders (Table S3). Pain impacted on daily function with an average BPI interference score of 5.2 ± 3.06 out of 10.

**Table 2.** Pain characteristics.

	*n	
Pain VAS (median, IQR)	100	68 [38, 81]
BPI Average Severity Score (mean ± SD)	63	5.18, 2.53
BPI Interference Score	59	5.2 ± 3.06
EQ5D3L pain (n, %)	100	
Severe		35, 35%
Moderate		55, 55%
None		10, 10%
Widespread pain (n, %)	101	47, 46.5%
WPI Score* ≥7	100	44, 44%
WPI Score* <7	100	56, 56%
Back pain (n, %)	101	70, 69.3%
Fibromyalgia (n, %)	101	36, 35.6%

The results of the baseline pain questionnaires are summarised in this table outlining the pain characteristics of this cohort.

*n: number of participants with complete data; VAS: Visual Analogue Scale; BPI: Brief Pain Inventory; WPI: Widespread pain index.

Table 3 compares pain, fatigue severity, and FMS prevalence by disease activity at baseline. In the high BS disease activity group, 76% reported severe pain (PVAS >70) compared to 39.1% in the low disease activity group (*p* = .003). Prevalence of widespread pain was higher in those with high BS disease activity (72%) than in those with low disease activity (37.7%) (*p* = .003). Fatigue severity did not vary with BS disease activity (*p* = .07). Concomitant FMS diagnosis is more prevalent in the high disease activity group (52%) than the low disease activity group (29%) (*p* = .04).

**Table 3.** Pain, fatigue severity, and fibromyalgia syndrome prevalence by disease activity.

	Low disease activity (*n* = 69) (*n*, %)	High disease activity (*n* = 25) (*n*, %)
Pain severity		
Mild pain (PVAS < 40)	23, 33.3%	1, 4%
Moderate pain (PVAS < 40–70)	19, 27.5%	5, 20%
Severe pain (PVAS >70)	27, 39.1%	19, 76%
	X^2^(2, N = 94) = 11.69 *p* = .003	
Widespread pain	26, 37.7%	18, 72%
Localised pain	43, 62.3%	7, 28%
	X^2^(1, N = 94) = 8.68 *p* = .003	
WPI Score* ≥7	24, 35.3%	18, 72%
WPI Score* <7	44, 64.7%	7, 28%
	X^2^(1, N = 93) = 9.94 *p* = .002	
Fatigue severity		
Mild fatigue (FVAS < 40)	13, 18.8%	1, 4%
Moderate fatigue (FVAS < 40–70)	21, 30.4%	5, 20%
Severe fatigue (FVAS >70)	35, 50.7%	19, 76%
	X^2^(2, N = 94) = 5.48 *p* = .065	
BS	49, 71%	12, 48%
BS +FMS	20, 29%	13, 52%
	X^2^(1, N = 94) = 4.27 *p* = .04	

Categorical variables were created from the pain visual analogue scale (PVAS) and the fatigue visual analogue scale (FVAS). Mild, moderate, and severe were defined as < 40, 40–70 and >70, respectively, for both fatigue and pain. High and low disease activity were defined as a Behçet’s disease current activity form (BDCAF) score ≥4 or <4, respectively.

VAS: Visual Analogue Scale; WPI: Widespread pain index; BS: Behçet’s Syndrome; FMS: Fibromyalgia syndrome.

*WPI data missing for one patient in low disease activity group.

**Figure 1.**
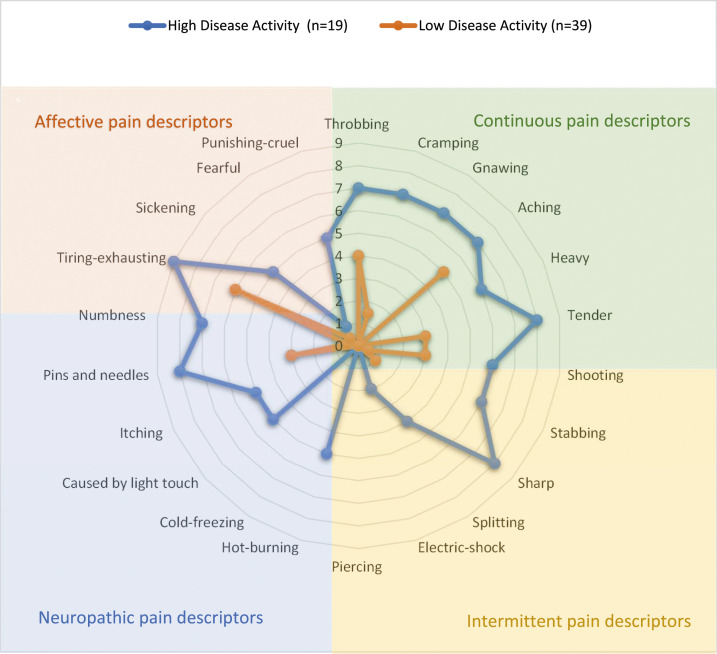
Descriptors of pain from the Short Form McGill questionnaire stratified by disease activity. 22 pain descriptors are rated 0 equal to no pain and 10 equal to the worst pain ever during the past week. The median value for each descriptor is plotted. The pain descriptors are divided into categories (colour coded): affective, continuous, neuropathic, and intermittent. High and low disease activity were defined as a Behçet’s disease current activity form (BDCAF) score ≥4 or <4, respectively.

The most reported pain descriptors were within the continuous group, Figure 1. The intensity of these terms, rated out of 10, is higher in the high disease activity group.

Table 4 compares baseline pain and fatigue between BS participants with and without a diagnosis of FMS. BS participants with comorbid FMS report higher fatigue severity (*p* = .004), pain severity (*p* = .003), and frequency of widespread pain (*p* = .003) compared to those with BS alone. Participants with FMS report higher BDCAF scores despite removal of components relating to joint symptoms (mBDCAF). The mean mBDCAF of participants with FMS was 2.09 ± 1.3 compared to 1.53 ± 1.3 in participants without FMS (*p* = .047).

**Table 4.** Pain and fatigue by concomitant fibromyalgia syndrome diagnosis.

	BS (*n* = 65) (*n*, %)	BS + FMS (*n* = 36) (*n*, %)
Pain severity*		
Mild (PVAS < 40)	23, 35.9%	3, 8.3%
Moderate pain (PVAS < 40–70)	17, 26.6%	8, 22.2%
Severe pain (PVAS >70)	24, 37.5%	25, 69.4%
	X^2^(2, N = 100) = 11.73 *p* = .003	
Widespread pain	23, 35.4%	24, 66.7%
Localised pain	42, 64.6%	12, 33.3%
	X^2^(2, N = 101) = 9.11 *p* = .003	
WPI Score* ≥7	22, 33.8%	22, 62.9%
WPI Score* <7	43, 66.2%	13, 37.1%
	X^2^(2, N = 100) = 7.77 *p* = .05	
Fatigue severity		
Mild fatigue (FVAS < 40)	15, 23.1%	0
Moderate fatigue (FVAS < 40–70)	18, 27.7%	9, 25%
Severe fatigue (FVAS >70)	32, 49.2%	27, 75%
	X^2^(2, N = 101) = 11 *p* = .004	

Comparison of baseline pain and fatigue between participants with Behçet’s syndrome (BS) and participants with BS and Fibromyalgia syndrome (FMS).

VAS: Visual Analogue Scale; WPI: Widespread pain index; BS: Behçet’s Syndrome; FMS: Fibromyalgia syndrome.

*Pain Severity data missing for one patient in BS group and WPI data missing for one patient in the BS + FMS group.

**Figure 2.**
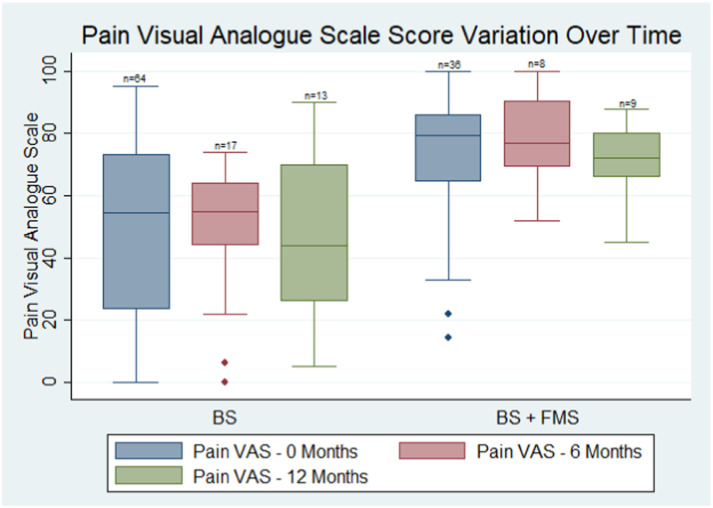
Comparison of pain visual analogue scores over time between Behcet’s syndrome patients with a diagnosis of fibromyalgia syndrome (BS + FMS) and without (BS). Pain VAS Scores are compared at baseline, 6 months, and 12 months prospectively between the two groups: BS: Behçet’s Syndrome, BS +FMS: Comorbid Fibromyalgia syndrome.

Unfortunately, prospective data were limited due to loss to follow-up. 25 participants completed questionnaires at 6 months and 22 participants completed questionnaires at 12 months. Pain VAS scores were compared at 0, 6, and 12 months (Figure 2). The change in pain VAS prospectively was very heterogeneous. No clear trend was observed in pain VAS in the small subgroup with longitudinal measurement. Variation in pain scores over time did not differ from those without FMS, however, the median pain VAS scores remained higher in the comorbid FMS group over the 12 months.

## Discussion

There is a scarcity of research describing pain in BS and there is conflicting evidence assessing the relationship between disease activity and pain. To our knowledge, this is the first study describing the pain experience of UK BS patients and exploring the relationship with disease activity. Our BS cohort had female predominance, in keeping with studies from America and in contrast to the male predominance classically found on the ‘Silk Road’ geographical areas. These may be due to phenotypic differences with BS geographically or due to greater female engagement with health services in western countries.^28^

Our findings highlight a large burden of pain in this cohort. 90% reported pain; 35% reporting severe pain, and 55% reporting moderate pain. The median pain VAS was 68. This is high in comparison to other rheumatological conditions. The average pain VAS has been reported as 23 in Systemic Lupus Erythematosus^29^ and 34 in Rheumatoid Arthritis (RA).^30^ Conversely, Moses Alder et al. compared pain scores between patients with BS and RA and found they reported similar levels of pain. However, the BS patients were on treatment and the RA patients were not, therefore, the pain scores of untreated BS patients are likely worse than represented in this study.^9^ On the SF-MPQ-2 questionnaire, Figure 1, participants selected more continuous pain terms to describe their pain than other subgroups of descriptors. This implies the pain experience is chronic in nature compared to the relapsing remitting nature known of BS.

Our study supports existing literature that FMS prevalence in BS ranges between 5.7% and 37.1%, the prevalence in our cohort was 35.6%. Wolfe at al. revised the chronic widespread pain (CWP) criteria, proposing that combining the ACR 2016 definition of widespread pain with a WPI ≥7 better describes CWP.^26^ We found 46.5% of participants experienced widespread pain, of which 91.3% had a WPI Score ≥7. The proportion of participants reporting CWP is higher than the prevalence of clinically diagnosed FMS; This poses the question whether fibromyalgia is underdiagnosed in this cohort or whether CWP is a feature of BS.

Alongside pain, fatigue and psychological distress are prevalent in this cohort. Most participants experienced severe fatigue (76%) with an average fatigue VAS of 66/100. Self-reported depression was far lower (38.6%) than when tested formally using the PHQ-9 (86.4%). This discrepancy highlights the importance of assessing psychological wellbeing in BS. Psychological well-being has a bi-directional effect on chronic pain: anxiety/depression can be the result of chronic pain but they also predispose patients to chronic pain.^31^ Equally, higher rates of depression/anxiety are often found in patients with chronic rare diseases.^32^ Patients with BS experience long delays to diagnosis due to the wide spectrum of symptoms, lack of biomarkers, and low clinical suspicion due to rarity.^33^ It is proposed that delay to diagnosis worsens feelings of anxiety.

BS patients experience poor quality of life, rating the BDQOL 15.4 ± 9.4 and the EQVAS 48 ± 25.4. This is low compared to the UK population where the index value for the EQVAS is 82.8, (100 being the best imaginable state and 0 being the worst).^34^

We explored the relationship between disease activity and pain. Pain is more widespread and more intense in those with high disease activity; 76% reported severe pain in the high activity group compared to 39.1% in the low disease activity group (*p* = .003). Although the low disease activity group experience less severe pain than the high disease activity group, the burden remains high at nearly 40% experiencing severe pain. The proportion of participants with FMS was higher in the high disease activity group (52%), BDCAF ≥4, compared to the low disease activity group (29%), BDCAF <4 (*p* = .04). A secondary analysis removing the arthralgia and arthritis components of the BDCAF found the disease activity of BS remained significantly higher in the comorbid FMS cohort (2.09 ± 1.3 vs 1.53 ± 1.3, *p* = .047).

Our finding’s supports Ayar et al.’s conclusion that FMS is related to disease activity in patients with BS.^14^ Haliloglu et al. also found the average BDCAF of BS patients with FMS, although a small sample (*n* = 3), to be statistically higher than those with BS alone (*n* = 50).^35^

However, Melikoglu et al. found that despite similar objective manifestation of BS, patients with FMS had worse impression of disease activity than those with BS alone.^15^ This emphasizes the potential role of reporting bias. The DAS28 scores of RA patients with comorbid FMS have been found to be significantly higher than those with RA alone.^36^ Co-morbid FMS may influence the interpretation of BDCAF scores.

Our findings raise the question – ‘Is there a unifying pathophysiology between FMS and BS?’ BS is theorised to have characteristics of both autoinflammatory and autoimmune conditions with activation of both the innate and adaptive immune system.^3^The opinion that FMS is non-immune mediated has changed with recent studies.^37,38^ Goebel et al. have recently shown that IgG from patients with FMS sensitized nociceptive neurons in mice producing sensory hypersensitivity, this strongly implicates antibody-dependent processes in the pathophysiology of FMS.^39^ Additionally, an imbalance of cytokines is assumed to play a role in the initiation and maintenance of pain. A systematic review and meta-analysis of cytokines in FMS showed that patients with FMS have higher plasma levels of IL-6 and IL-8 than healthy controls.^40–42^ These cytokines are also elevated in BS and IL-6 levels have been correlated with arthritic manifestations.^43–46^ Further research is needed to investigate whether comorbid FMS contributes to inflammation in BS, worsening disease activity.

In contrast, Fitzcharles et al.^47^ explored the concept of comorbid FMS. They theorised that patients with rheumatological conditions may develop a ‘pain prone phenotype’ where existing pain initiates central sensitization resulting in greater pain severity and widespread location. Equally, biopsychosocial factors have great impact on pain and are strongly associated with FMS. The high prevalence of psychological distress in the BS cohort may contribute to the high prevalence of FMS; however, this would not explain the relation to disease activity. There is complexity in evaluating the interplay between FMS and BS as the pathophysiology of either is not fully understood.

To our knowledge, this study is the largest study evaluating fibromyalgia frequency and its relationship to BS disease activity worldwide. This is a population-based study which is generalisable to the UK BS cohort as patients attend LBC from across the UK. There are many limitations to this study. When assessing disease activity, we did not measure laboratory markers of inflammation or record factors that can contribute to levels of inflammation such as body mass index or smoking status. We did not collect data on sleep and therefore were unable to apply the full ACR 2016 diagnostic criteria for FMS. FMS prevalence was determined from consultant diagnosis. There may be variability between clinicians’ propensity to diagnose comorbid FMS. Equally, participants may have acquired a diagnosis of FMS, on their path to a diagnosis of BS, which may no longer be applicable. Our study includes a limited number of participants and studies with larger sample sizes are needed to confirm our findings, however, may be difficult due to the nature of rare diseases.

In conclusion, we have demonstrated that the UK BS population suffer a large burden of pain, fatigue, and psychological issues. The majority of BS patients experience moderate to severe CWP. Pain intensity and comorbid FMS diagnosis are related to worse disease activity. These findings highlight the importance of pain management strategies for patients with BS. They also highlight the potential role of disease modifying drugs in treating pain. This is a hypothesis generating paper which poses new questions into the research of pain in BS and comorbid FMS. Further studies are required to explore the mechanism of CWP in BS and the correlation with disease activity.

## Supplemental Material

Supplemental Material - Characteristics of pain and their relationship to disease activity in UK patients with Behçet’s syndrome: a prospective cohort study Click here for additional data file. Supplemental Material for Characteristics of pain and their relationship to disease activity in UK patients with Behçet’s syndrome: a prospective cohort study by K Plant, A Goebel, J Nair, R Moots, Laura Chadwick and N Goodson in British Journal of Pain
