# Supplementary material for: Characteristics of pain and their relationship to disease activity in UK patients with Behçet’s syndrome: a prospective cohort study
Source: Br J Pain. 2023 Aug 29;17(6):560–8. doi: 10.1177/20494637231198200 (PMC10642501; doi:10.1177/20494637231198200)
Supplement: Supplemental Material - Characteristics of pain and their relationship to disease activity in UK patients with Behçet’s syndrome: a prospective cohort study [file sj-pdf-1-bjp-10.1177_20494637231198200.pdf]

**TableS1 - Questionnaires**	
Pain Visual Analogue Scale<br><br>***Fatigue Visual analogue scale***	The VAS is a straight horizontal line - 100 mm. The ends are defined as 'no pain', and the right end 'extreme pain' The patient marks along this line which is transferred to a numerical number. This number can be converted to a pain severity category using the following cut off values:<br>0-39.99 Mild pain/fatigue<br>40-69.99 Moderate pain/ fatigue<br>70-100 Severe pain/fatigue(1)
EQ-5D-3L	The EQ-5D-3L consists of 5 questions covering the following domains: mobility, self-care, usual activity, pain and anxiety/depression. Patients rate each domain either no problem, some problem or extreme problem. The questionnaire also includes an EQ VAS: patients rate their general state of health, 0 being worst to 100 being the best(2).
Behcet's disease quality of Life questionnaire (BDQOL)	Patients mark true (=1) or false (=0) next to 30 statements which describe how BS negatively effects their life(3).
Generalised anxiety disorder scale 7	The GAD-7 assesses the frequency that seven symptoms of anxiety occurred over the last two weeks by using a scale from 0 (*not at all*) to 3 (*nearly every day*). Higher scores denote more severe symptoms of anxiety. Scores of 5, 10, 15 were the cut-off point for mild, moderate and severe(4).
Patient health questionnaire – 9 (PHQ-9)	The PHQ-9 assesses the presence of 9 depressive symptoms over a 2 week period by using a scale from 0 *(not at all)* to 3 *(nearly every day).*<br>The higher the score the more severe the depression. The cut off points for mild, moderate, moderately severe and severe are 5, 10, 15, 20 respectively(5).
Behçet's disease activity form (BDCAF)	Assesses the presence of 12 clinical features of BD over the last 4 weeks: Headache, Mouth Ulceration, Genital Ulceration, Erythema Nodosum, Skin Pustules, Arthralgia, Arthritis, Nausea/Vomiting, Diarrhoea, New eye involvement, New central nervous system involvement, New major vessel inflammation. Each clinical feature selected scores as 1 point(6).
Pain Mannequin	The patient shades in sites of pain on the blank body mannequin(1).
Brief pain inventory	The BPI assesses the severity of pain and the impact of pain on daily functions. The patients rate their worse pain, their least pain, their current pain and their average pain over 24 hours out of 10. This combined score is averaged to create the BPI severity score. There are 7 statements of daily function. The patients rate out of 10 how much their pain has interfered with each statement within the last 24 hours. The combined score is averaged to create the BPI interference score.
Short form McGill Pain Questionnaire 2	22 pain descriptors are rated using numerical rating score 0-10(7).<br>The pain descriptors are divided into 4 subscales: continuous (6 items), intermittent (6 items), neuropathic (6 items) and affective (4 items).

**TableS2 - Assessment of psychological wellbeing**			
**GAD-7 (n, %)**	All (n =65)	Female (n =48)	Male (n =17)
None	15, 23.1%	9, 18.8%	6, 35.3%
Mild (≥ 5)	17, 26.2%	12, 25%	5, 29.4%
Moderate (≥ 10)	17, 26.2%	16, 33.3%	1, 5.9%
Severe (≥ 15)	16, 24.6%	11, 22.9%	5, 29.4%
**PHQ-9 (n, %)**	**All** (n =66)	**Female** (n =49)	**Male** (n =17)
None	9, 13.6%	4, 8.2%	5, 29.4%
Mild (≥ 5)	13, 19.7%	10, 20.4%	3, 17.7%
Moderate (≥ 10)	13, 19.7%	11, 22.5%	2, 11.8%
Moderately severe (≥ 15)	18, 27.3%	15, 30.6%	3, 17.7%
Severe (≥ 20)	13, 19.7%	9, 18.4%	4, 23.5%
Anxiety (GAD-7) and Depression (PHQ-9) scores across the cohort outlined above.			

**TableS3- Disease Activity and Pain Characteristics by Gender**	**Gender**	
**Disease Activity**	**Female (n =71)**	**Male (n =23)**
High disease activity (n,%)	19, 26.8%	6, 26.1%
Low disease activity (n,%)	52, 73.2%	17, 73.9%
	X^2(1, N=94) = 0.00 P=0.949	
**Pain characteristics**	**(n =75)**	**(n =25)**
EQ5D3L Pain - Severe (n,%)	26, 34.7%	9, 36%
EQ5D3L Pain - Moderate (n,%)	41, 54.7%	14, 56%
EQ5D3L Pain - None (n,%)	8, 10.7%	2, 8%
	X^2(2, N=100) = 0.15 P=0.928	
Pain Visual Analogue Scale (VAS) (median + IQR)	68 [41, 81]	66 [29, 79]
	**(n=75)**	**(n=26)**
Generalised pain (n,%)	36, 48%	11,42.3%
	X^2(1, N=101) = 0.25 P=0.616	
Back pain (n,%)	52, 69.3%	18, 69.2%
	X^2(1, N=101) = 0.00 P=0.992	
Fibromyalgia (n,%)	30, 40%	6, 23.1%
	X^2(1, N=101) = 2.41 P= 0.121	
Widespread Pain Index (median, IQR)	**(n=74)**	**(n=26)**
	5.5 [3, 9]	4.5, [1,7]
The table outlines disease activity and pain characteristics compared between genders. High and low disease activity were defined as a Behçet's disease current activity form (BDCAF) score ≥ 4 or < 4 respectively		

1. Hawker GA, Mian S, Kendzerska T, French M. Measures of adult pain: Visual Analog Scale for Pain (VAS Pain), Numeric Rating Scale for Pain (NRS Pain), McGill Pain Questionnaire (MPQ), Short-Form McGill Pain Questionnaire (SF-MPQ), Chronic Pain Grade Scale (CPGS), Short Form-36 Bodily Pain Scale (SF-36 BPS), and Measure of Intermittent and Constant Osteoarthritis Pain (ICOAP). Arthritis Care Res (Hoboken). 2011;63 Suppl 11:S240-52.
2. Bhadhuri A, Kind P, Salari P, Jungo KT, Boland B, Byrne S, et al. Measurement properties of EQ-5D-3L and EQ-5D-5L in recording self-reported health status in older patients with substantial multimorbidity and polypharmacy. Health and Quality of Life Outcomes. 2020;18(1):317.
3. Gilworth G, Chamberlain MA, Bhakta B, Haskard D, Silman A, Tennant A. Development of the BD-QoL: a quality of life measure specific to Behçet's disease. The Journal of Rheumatology. 2004;31(5):931.
4. Doi S, Ito M, Takebayashi Y, Muramatsu K, Horikoshi M. Factorial Validity and Invariance of the 7-Item Generalized Anxiety Disorder Scale (GAD-7) Among Populations With and Without Self-Reported Psychiatric Diagnostic Status. Frontiers in psychology. 2018;9:1741-.
5. Kroenke K, Spitzer RL, Williams JB. The PHQ-9: validity of a brief depression severity measure. Journal of general internal medicine. 2001;16(9):606-13.
6. Lawton G, Bhakta BB, Chamberlain MA, Tennant A. The Behcet's disease activity index. Rheumatology (Oxford). 2004;43(1):73-8.
7. Dworkin RH, Turk DC, Revicki DA, Harding G, Coyne KS, Peirce-Sandner S, et al. Development and initial validation of an expanded and revised version of the Short-form McGill Pain Questionnaire (SF-MPQ-2). Pain. 2009;144(1-2):35-42.
